# Investigating the Perovskite Ag_1-3x_La_x_NbO_3_ as a High-Rate Negative Electrode for Li-Ion Batteries

**DOI:** 10.3389/fchem.2022.873783

**Published:** 2022-04-13

**Authors:** Etienne Le Calvez, Julio César Espinosa-Angeles, Grace J. Whang, Nicolas Dupré, Bruce S. Dunn, Olivier Crosnier, Thierry Brousse

**Affiliations:** ^1^ Nantes Université, CNRS, Institut des Matériaux de Nantes Jean Rouxel, IMN, Nantes, France; ^2^ Réseau sur le Stockage Electrochimique de l’Energie (RS2E), CNRS FR 3459, Amiens Cedex, France; ^3^ Department of Materials Science and Engineering, University of California, Los Angeles, Los Angeles, CA, United States

**Keywords:** silver niobate, lithium-ion battery, high rate anode material, fast charging, innovative oxide

## Abstract

The broader development of the electric car for tomorrow’s mobility requires the emergence of new fast-charging negative electrode materials to replace graphite in Li-ion batteries. In this area, the design of new compounds using innovative approaches could be the key to discovering new negative electrode materials that allow for faster charging and discharging processes. Here, we present a partially substituted AgNbO_3_ perovskite material by introducing lanthanum in the A-site. By creating two vacancies for every lanthanum introduced in the structure, the resulting general formula becomes Ag_1-3x_La_x_□_2x_NbO_3_ (with x ≤ 0.20 and where □ is a A-site vacancy), allowing the insertion of lithium ions. The highly substituted Ag_0.40_La_0.20_□_0.40_NbO_3_ oxide shows a specific capacity of 40 mAh.g^−1^ at a low sweep rate (0.1 mV s^−1^). Interestingly, Ag_0.70_La_0.10_□_0.20_NbO_3_ retains 64% of its capacity at a very high sweep rate (50 mV s^−1^) and about 95% after 800 cycles. *Ex situ* ^7^Li MAS NMR experiments confirmed the insertion of lithium ions in these materials. A kinetic analysis of Ag_1-3x_La_x_□_2x_NbO_3_ underlines the ability to store charge without solid-state ion-diffusion limitations. Furthermore, *in situ* XRD indicates no structural modification of the compound when accommodating lithium ions, which can be considered as zero-strain material. This finding explains the interesting capacity retention observed after 800 cycles. This paper thus demonstrates an alternative approach to traditional insertion materials and identifies a different way to explore not-so common electrode materials for fast energy storage application.

## Graphical Abstract

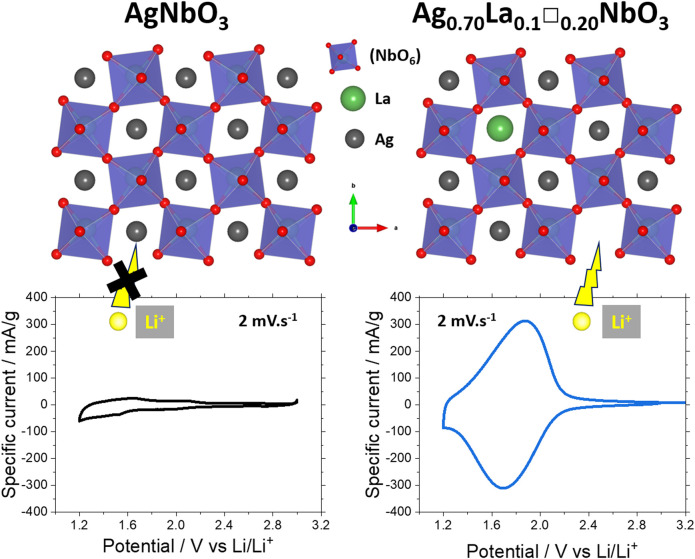

## Introduction

To eliminate our dependence on carbon-based energy production, specifically in the field of mobility, it is essential to develop energy storage devices able to store the intermittent energy harvested by renewable energy sources (Armand and Tarascon 2008). A key to the deployment of electric vehicles is the ability of batteries to charge quickly while ensuring long cycle life, and optimized safety (Burnham et al., 2017; Li et al., 2020). The current use of graphite as a negative electrode only partially satisfies these needs (Choi et al., 2020). In fast-charging batteries, graphite enables the insertion of lithium at an average potential close to that of Li^+^/Li (0.1 V vs. Li^+^/Li), which can lead to the formation of lithium plating followed by dendrite growth, resulting in possible short circuits and thermal runaway. Moreover, the specific capacity of graphite is drastically reduced at high current densities due to kinetic limitations on ion mobility between graphene layers (Sivakkumar et al., 2010).

For these reasons, alternative negative electrode materials have attracted increased attention over the past decade One of the best known negative electrode materials for high-rate applications is Li_4_Ti_5_O_12_ (LTO) (Prakash et al., 2010; Song et al., 2014; Thackeray and Amine 2021). Crystallizing in a spinel structure, this zero strain material offers an alternative route towards designing fast charging batteries with a relatively high specific capacity. Moreover, new approaches have been proposed to offer more options for the replacement of graphite electrode. Among others, shear-structure materials including TiNb_2_O_7_ (Griffith et al., 2019) or Nb_16_W_18_O_93_ (Griffith et al., 2018) offer relatively large capacity values at high rate between 1V and 3V vs*.* Li^+^/Li. Low volume changes upon lithium insertion and an operating potential that avoids the formation of lithium-consuming solid electrolyte interphase (SEI) are the main advantages offered by these materials. In addition, a new approach has recently been proposed to transform a material traditionally used as an ionic conductor into a material for high-rate Na-ion batteries. Partial substitution of Al by Fe in ß”-Al_2_O_3_ offers both ionic and electronic conduction, allowing fast and reversible Na^+^ intercalation (Butts et al., 2021). This material displays cyclic voltammogram with broad peaks attributed to a surface-controlled charge storage mechanism. Similar approaches are needed to unravel different fast charging materials and their associated mechanisms.

In this work, we propose an alternative way to produce materials that can insert alkali cations with minor diffusion limitation, thus allowing high current density operation. More precisely, the A-site of AgNbO_3_ perovskite synthesized by sol-gel process has been partially substituted by lanthanum (La^3+^), allowing the creation of vacancies in this crystallographic site. Insertion of Li ions is observed in every substituted Ag_1-3x_La_x_□_2x_NbO_3_ compound (with x > 0), thus leading to experimental specific capacities close to the theoretical values. Calculation of *b*-value shows a non-diffusion-controlled mechanism and *in situ* XRD shows negligible volume expansion (zero strain electrode) during the insertion/deinsertion mechanism for this new family of materials, therefore explaining the cycling stability of Ag_1-3x_La_x_□_2x_NbO_3_.

## Experimental Section

### Synthesis of Ag_1-3x_La_x_□_2x_NbO_3_

Ag_1-3x_La_x_□_2x_NbO_3_ powders were synthesized by a sol-gel route (SG) based on a previous report (Wu et al., 2013). Briefly, a stoichiometric mixture of NbCl_5_ (99%, Sigma Aldrich), La(NO_3_)_3_ (99.9%, Alfa Aesar), AgNO_3_ (99.9%, Alfa Aesar), and citric acid in excess (99+%, Alfa Aesar) was dissolved in 30% (w/w) H_2_O_2_ in H_2_O (Sigma Aldrich). Upon complete dissolution of the species, the solution turned to a dark yellow color. At this point, the solution was heated at 120°C until the formation of a dark brown gel. The latter was heated to 300°C overnight to remove organic moieties and dehydrate the gel. The resulting powder is ground in an agate mortar and then annealed at 650°C for 6 h in an alumina crucible to crystallize the material. Depending on the degree of substitution, the resulting powder ranges from dark grey for AgNbO_3_ to whitish powder in the case of Ag_0.40_La_0.20_□_0.40_NbO_3._

### Characterization

#### Powder X-Ray Diffraction

X-ray diffraction (XRD) patterns were collected using a PANalytical X’Pert Pro diffractometer (Malvern Panalytical, Almelo, Netherlands). An X’Celerator detector with Cu-Kα1-Kα2 (*λ* = 1.54060, 1.54439 Å) radiation was used with acceleration voltage and current of 40 kV and 40 mA, respectively. With a step scan of 0.0167°, diffraction patterns have been collected between 20° and 80° (2θ). Using pseudo-Voigt functions, Le Bail refinement was performed using FullProf Suite. Preliminary lattice parameter values have been taken from the literature (Sciau et al., 2004). For *in situ* XRD measurements, patterns were collected using a homemade “Leriche-like” cell (Leriche et al., 2010). During charge-discharge experiment, a current of 0.005 A g^−1^ was applied for 20 min and stopped during 30 min for XRD pattern acquisition.

#### Electron Microscopy and Energy Dispersive X-Ray Analyses

The morphology and particle size were studied with a transmission electron microscope ThermoFisher S/TEM Themis G3 at 300 kV (Breda) point to point resolution: 0.18 nm. SEM micrographs were obtained at 20 kV using a Zeiss MERLIN Instrument using in-Lens annular detector. Samples were prepared by dispersing a small fraction of the powder on a piece of conducting carbon tape. EDX was collected at 8.0 mm working distance using an X-Max 50 mm^2^ OXFORD Instruments detector. The measurements were performed on at least two samples per synthesis and for each sample at least 10 acquisitions were performed. The atomic percentage of each cation was determined with an accuracy of ±5%.

#### BET Measurement

The specific surface area of the powders was determined using the BET method (Brunauer-Emmett-Teller) from the 77 K nitrogen adsorption curves with a Quantachrome Nova 4200e equipment (Anton Paar).

#### Electrochemistry

Self-supported electrodes were fabricated by mixing, in a small amount of ethanol, 75% w/w Ag_1-3x_La_x_□_2x_NbO_3_, 15% w/w of conductive carbon (Carbon Black by Superior Graphite, Chicago, IL, United States) to increase electronic conductivity and 10% w/w poly (1,1,2,2-tetrafluoroethylene) dissolved in water (PTFE, Sigma Aldrich) to form a homogeneous paste. This mixture was homogenized in a mortar and then cold-rolled until electrodes with a mass loading of about 5–10 mg cm^−2^ were obtained. This level of loading enables reliable gravimetric values to be reported (Gogotsi and Simon 2011). Electrochemical measurements were performed using Swagelok cells. The positive electrode and metallic lithium (99.9%, Aldrich) were separated using a glass fiber membrane (70 mm, 10 mm diameter, GF/D Whatman) soaked with 1M LiPF_6_ dissolved in 1:1 %wt. EC/DMC (Solvionic, France, battery grade purity). The Swageloks were assembled in a glove box containing less than 0.1 ppm of O_2_ and H_2_O. A VMP3 potentiostat (from Biologic run under ECLab software version V11.36, Seyssinet-Pariset, France) was used for electrochemical measurements.

#### *Ex situ* MAS NMR

For MAS NMR analysis, free-standing electrodes with about 5–10 mg cm^−2^ were polarized for 10 h at different states of charge after performing a cyclic voltammogram (CV) at 0.1 mV s^−1^. After disassembling the Swageloks in a glove box, electrodes were cleaned with a few drops of DMC and then dried overnight under *vacuum* in a Büchi oven at 60°C. Then, the electrolyte-free electrode was placed in a 2.5 mm-diameter zirconia rotor for MAS NMR experiments. ^7^Li MAS NMR spectra were acquired on a Bruker Avance 200 spectrometer (B0 = 4.7 T, Larmor frequencies ν_0_ (^7^Li = 77.78 MHz) at RT. A Bruker MAS probe with a cylindrical 2.5 mm (o.d) zirconia rotor spun at a frequency of 30 kHz was used. Spectra were obtained by applying a single pulse sequence, and a recycle delay (D1) of 3s ensured the acquisition of quantitative spectra. ^7^Li integrated intensities were determined by using spectral simulation (DMFit Software) (Massiot et al., 2002). The resulting integrated intensities were normalized with respect to the mass of the sample contained in the NMR rotor, the number of scans, and the receiver gain. Due to low amount of lithiated active material used for the analyses, it was not possible to rely on ^6^Li MAS NMR to characterize local structural features.

## Results and Discussions

At room temperature, AgNbO_3_ crystallizes in an orthorhombic perovskite structure (space group Pbcm) with distorted [BO_6_] octahedra, similar to most perovskites (Sciau et al., 2004). Historically, AgNbO_3_ has been studied in material science as antiferroelectric material for energy storage applications. Substitutions in this lead-free material have been widely examined to stabilize the antiferroelectric phase and increase the power density. Various studies have explored the substitution of Ag^+^ in AgNbO_3_ by Li^+^ (Farid et al., 2019), Na^+^ (Takeda et al., 2003), and multivalent cations (Li et al., 2009; Juang et al., 2016; Gao et al., 2020) (Sr^2+^, La^3+^, Sm^3+^, etc.) to create A-site vacancies. All the substitutions by multivalent cations show an increase in the symmetry of the material until the presence of impurities at a high degree of substitution (Juang et al., 2016; Gao et al., 2019; Gao et al., 2020). In order to investigate the possible Li-ion insertion into A site vacancies of this perovskite, we synthesized La-doped AgNbO_3_ with different substitution ratios from x = 0 to x = 0.20. In a perovskite lattice, the coordination number of the A-site is 12, and 6 for the B-site. The ionic radius of silver is 1.54 Å, whereas that of niobium is 0.64 Å (Shannon and Prewitt 1969; Shannon 1976). Since the ionic radius of La for a coordination number of 12 is 1.36 Å, the silver-to-lanthanum substitution should be effective in the A site of the perovskite Ag_1-3x_La_x_□_2x_NbO_3_, where □ depicts a cation, vacancy in the A site of the perovskite, following reaction Eq. 1:

$$La_{2}O_{3}\to \;2La_{Ag}^{\cdot \cdot }+3O_{o}^{x}+4V_{Ag}^{\prime }$$ (1)

**FIGURE 1:**
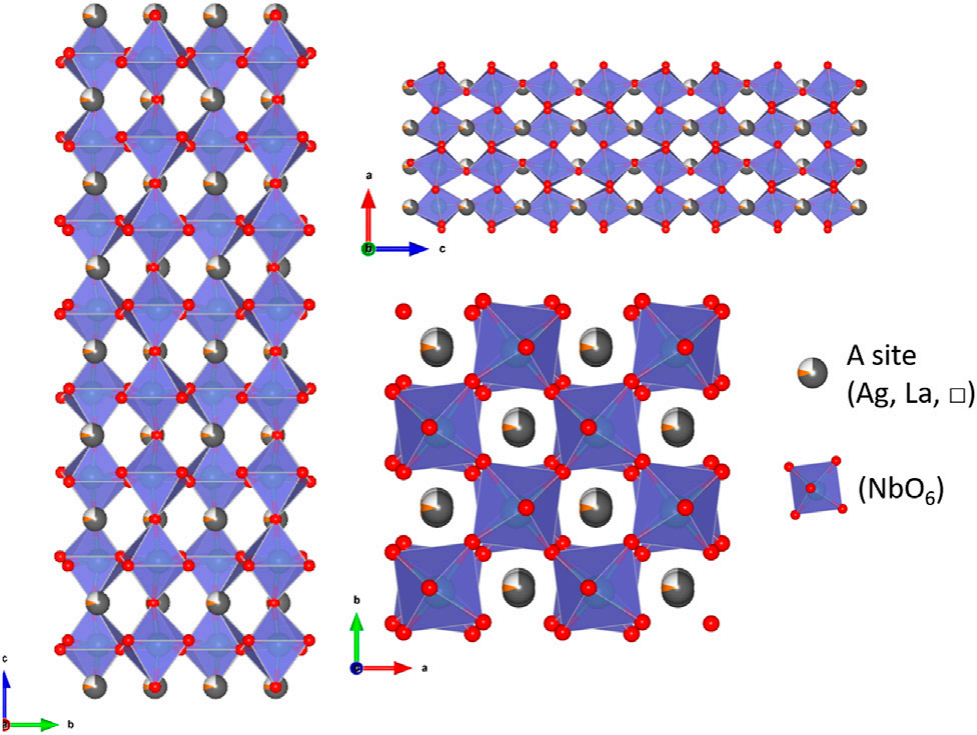
(A-B-C) respectively a, b, and c axis view of Ag_0.70_La_0.10_□_0.20_NbO_3_.

Here, we will name the samples Ag_0.85_La_0.05_□_0.10_NbO_3_, Ag_0.70_La_0.10_□_0.20_NbO_3_, Ag_0.55_La_0.15_□_0.30_NbO_3_ and Ag_0.40_La_0.20_□_0.40_NbO_3,_ respectively for x = 0.05, x = 0.10, x = 0.15 and x = 0.20. Figure 1 represents the crystallographic arrangement of Ag_0.70_La_0.10_□_0.20_NbO_3_ which shows both distortion of [NbO_6_] octahedra and the partial substitution of Ag by La in A-site with the corresponding vacancies.

**FIGURE 2:**
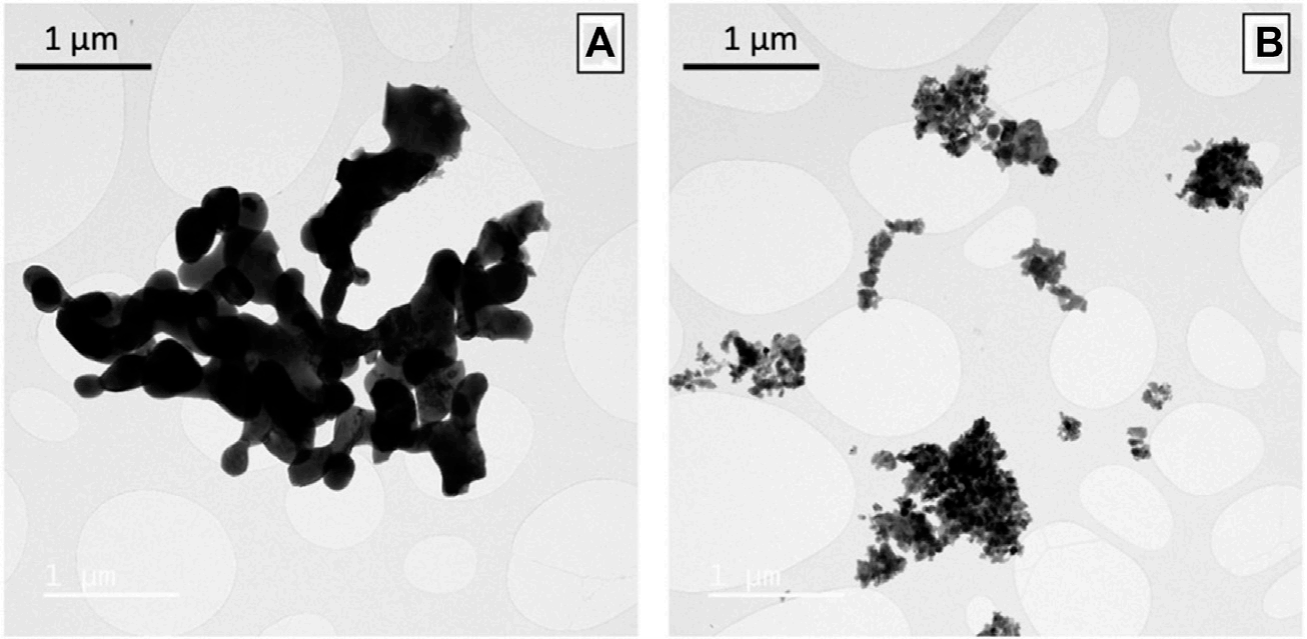
TEM images of **(A)** AgNbO_3_ and **(B)** Ag_0.70_La_0.10_□_0.20_NbO_3_ after annealing at 650°C showing the difference in particles size between unsubstituted and substituted oxides.

The synthesis of Ag_1-3x_La_x_□_2x_NbO_3_ by a ceramic route was difficult to achieve. One reason is the reduction of Ag_2_O to metallic Ag for a temperature higher than 150°C (Chun et al., 2009; Shin et al., 2013). A second reason is the thermodynamic competition between AgNbO_3_ and Ag_2_Nb_4_O_11_ does not allow one to easily obtain a phase pure material, even by adding an excess of Ag_2_O (Valant et al., 2007). For this reason, the different stoichiometries of AgNbO_3_ were synthesized by a sol-gel (SG) process which has been demonstrated to be effective for the synthesis of perovskite materials (Huang et al., 1996; Y.; Li et al., 2010; Huízar-Félix et al., 2012). Moreover, the heat treatment at lower temperature (650°C) of the amorphous AgNbO_3_ particles will avoid the reduction of silver. Figure 2A,B shows both AgNbO_3_ and Ag_0.70_La_0.10_□_0.20_NbO_3_ agglomerated nanoparticles which are typical morphologies of sol-gel synthesis. AgNbO_3_ particles are larger than those of Ag_0.70_La_0.10_□_0.20_NbO_3_, a feature confirmed by nitrogen adsorption measurements (BET method; Supplementary Table S1). These measurements revealed a specific surface area of 3 m^2^ g^−1^ for AgNbO_3_ and 13 m^2^ g^−1^ for Ag_0.70_La_0.10_□_0.20_NbO_3_. In addition, as shown in Supplementary Table S2, EDX cationic ratio between Ag, La and Nb for every Ag_1-3x_La_x_□_2x_NbO_3_ confirms the presence of vacancies in all the powders of La-substituted AgNbO_3_.

**FIGURE 3:**
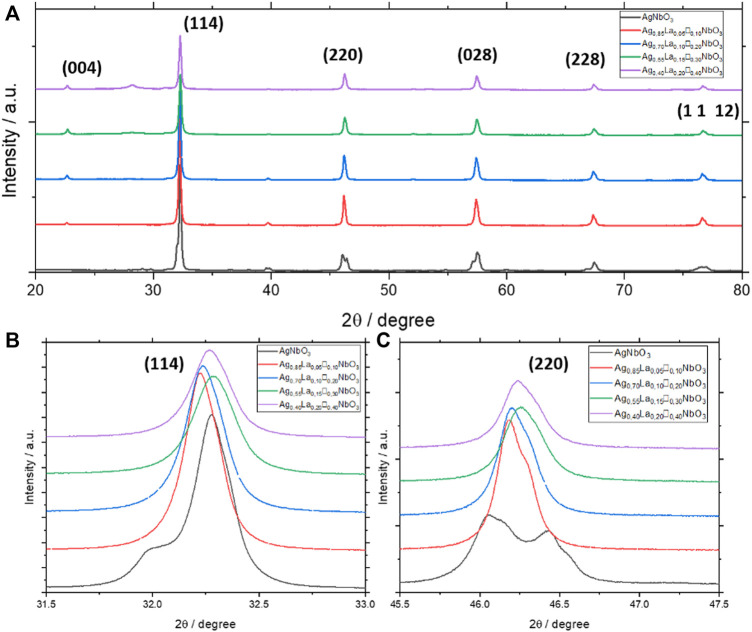
**(A–C)** XRD patterns of Ag_1-3x_La_x_□_2x_NbO_3_ for different value of x.

XRD was used to better understand the structural differences among all Ag_1-3x_La_x_□_2x_NbO_3_ powders. As shown in Figure 3A., AgNbO_3_ has a perovskite structure with no secondary phase, although splitting of several peaks is observed. Ag_0.85_La_0.05_□_0.10_NbO_3_ (x = 0.05) and Ag_0.70_La_0.10_□_0.20_NbO_3_ (x = 0.10) also show the perovskite structure with no secondary phase visible in the diffractograms. However, starting from x = 0.15, impurity peaks appear, perhaps due to the lack of cations on the A-site, resulting in collapse of the structure. This is in agreement with previous reports on the A-site substitution in AgNbO_3_ (Li et al., 2009; Juang et al., 2016). Depending on the size of the dopant cation, the perovskite structure can accept up to a certain amount of substitution, sometimes accompanied by the appearance of extra peaks which correspond to secondary phases. The decrease in peak intensity for Ag_0.55_La_0.15_□_0.30_NbO_3_ and Ag_0.40_La_0.20_□_0.40_NbO_3_, compared to other samples, is an indicator of the stress caused by the high proportion of La in the perovskite. For the substituted oxides, a decrease in peak splitting was observed with increasing lanthanum substitution (Figures 3B–C). Further, the disappearance of peak splitting by x = 0.15 suggests a higher degree of symmetry in the substituted materials due to lower [NbO_6_] octahedra distortion.

To get greater insight into the structure of the synthesized materials, Le Bail structural refinements were performed for each composition. First, as indicated in Supplementary Figure S1, the refinement of AgNbO_3_ confirms that the Pbcm space group well represents the structure of AgNbO_3_. Cell parameters of AgNbO_3_ were extracted from the refinement as shown in Supplementary Figure S2 and Supplementary Table S3. We found that the cell parameters and the cell volume increase with increasing substitution of La for Ag_1-3x_La_x_□_2x_NbO_3_, which is in agreement with a previous report (Li et al., 2009). Moreover, this is consistent with a decrease of NbO_6_ octahedra distortions previously described above. Refinements using a higher symmetry space group (Pm3m) have been performed to check that no phase transition appears with the substitution of silver by lanthanum. However, they did not lead to conclusive results. For this reason, we assume that the same space group (Pbcm) can be used to describe the crystallographic structure of all the Ag_1-3x_La_x_□_2x_NbO_3_ powders.

**FIGURE 4:**
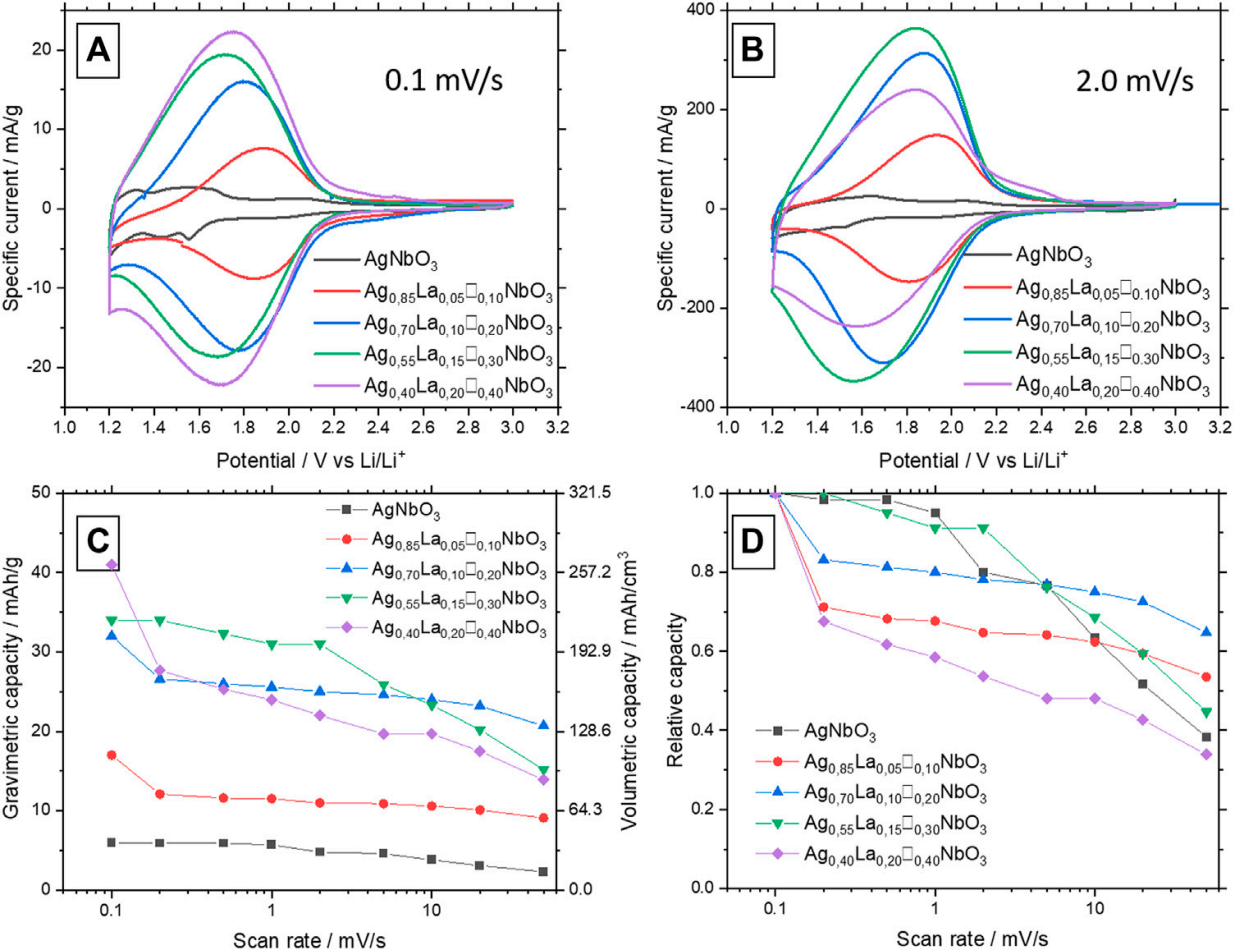
**(A)** CV at 0.1 mV s^−1^ between 1.2 and 3 V of Ag_1-3x_La_x_□_2x_NbO_3_ for different value of x. **(B)** CV at 2.0 mV s^−1^ between 1.2 and 3 V of Ag_1-3x_La_x_□_2x_NbO_3_ for different value of x. **(C)** Specific capacity of Ag_1-3x_La_x_□_2x_NbO_3_ for different value of x in function of the scan rate. **(D)** Relative capacity of Ag_1-3x_La_x_□_2x_NbO_3_ for different value of x in function of the scan rate.

To characterize the ability of this family of materials to reversibly store lithium ions, cyclic voltammetry at 0.1 mV s^−1^ was first performed. Note that this sweep rate should be considered as a low rate for power applications (around 5.5 h of charge). As depicted in Figure 4A, AgNbO_3_ does not show any electrochemical activity between 1.2 and 3.0 V vs. *Li* ^*+*^ */Li* at the selected scan rate*.* A negligible specific capacity of 6 mAh.g^−1^ is reported. However, for each substituted material, broad and reversible peaks appear between 2.2 and 1.2 V vs*. Li* ^*+*^ */Li*, characteristic of lithium insertion into the host structure. Considering no insertion is visible for the unsubstituted oxide, we propose the following reaction Eq. 2 to describe the present electrochemical reaction.

$$Ag_{1-3x}La_{x}{□}_{2x}\text{Nb}O_{3}+yLi_{x}+ye^{-}\to \;Li_{y}Ag_{1-3x}La_{x}{□}_{2x}\text{Nb}O_{3}$$ (2)

At 0.1 mV s^−1^, the specific capacity increases with the number of vacancies. During the first cycle at 0.1 mV s^−1^, specific capacities are close to the theoretical capacity considering the insertion of one lithium ion per vacancy: 16 mAh g^−1^, 35 mAh g^−1^, 54 mAh g^−1^, 76 mAh g^−1^ for Ag_0.85_La_0.05_□_0.10_NbO_3_, Ag_0.70_La_0.10_□_0.20_NbO_3_, Ag_0.55_La_0.15_□_0.30_NbO_3_, Ag_0.40_La_0.20_□_0.40_NbO_3_, respectively. Considering only the mass of active materials the gravimetric capacity is found to be equal to 38 mAh cm^−3^, 109 mAh cm^−3^, 206 mAh cm^−3^, 219 mAh cm^−3^ and 264 mAh cm^−3^ for AgNbO_3_, Ag_0.85_La_0.05_□_0.10_NbO_3_, Ag_0.70_La_0.10_□_0.20_NbO_3_, Ag_0.55_La_0.15_□_0.30_NbO_3_, Ag_0.40_La_0.20_□_0.40_NbO_3_, respectively. If the entire volume of the practical electrode is now considered (porosity volume ∼70%), the gravimetric capacity is found to be equal to 12 mAh cm^−3^, 32 mAh cm^−3^, 62 mAh cm^−3^, 66 mAh cm^−3^ and 79 mAh cm^−3^ for AgNbO_3_, Ag_0.85_La_0.05_□_0.10_NbO_3_, Ag_0.70_La_0.10_□_0.20_NbO_3_, Ag_0.55_La_0.15_□_0.30_NbO_3_, Ag_0.40_La_0.20_□_0.40_NbO_3_, respectively. Interestingly, no reduction of silver is observed in these materials indicating the strong interaction of silver cations within the perovskite structure, contrary to most silver-based materials reported in the literature, i.e., silver vanadates (West and Crespi 1995; Marschilok et al., 2010; Sauvage et al., 2010).

Cyclic voltammetry was used to characterize the performance of the lanthanum-substituted oxides at a higher sweep rate of 2 mV s^−1^ (Figure 4B). This sweep rate corresponds to 15 min of charge/discharge, which is consistent with the current goal for the development of electric vehicles (Colclasure et al., 2019). As was observed at 0.1 mV s^−1^, the CVs shown at 2 mV s^−1^ present very broad and reversible redox peaks.

As expected, the capacity of AgNbO_3_ remains very low compared to the substituted oxides. Its capacity is 5 mA h.g^−1^. Ag_0.85_La_0.05_□_0.10_NbO_3_ is the least substituted material and the one with the lower capacity at both 0.1 mV s^−1^ and 2 mV s^−1^ (32 mAh g^−1^). Interestingly, at 2 mV s^−1^, Ag_0.40_La_0.20_□_0.40_NbO_3_ has a lower capacity than Ag_0.70_La_0.10_□_0.20_NbO_3_ and Ag_0.55_La_0.15_□_0.30_NbO_3_, even though Ag_0.40_La_0.20_□_0.40_NbO_3_ shows the higher theoretical capacity most likely attributed to the higher amount of vacancies per unit cell. Such a decrease in capacity is explained by the lower degree of crystallinity which does not allow adequate diffusion of lithium ions in this substituted oxide. Moreover, the defects detected by the presence of peaks that did not belong to the orthorhombic lattice provides another possible explanation to the lower capacity of Ag_0.40_La_0.20_□_0.40_NbO_3_ at 2 mV s^−1^. In addition, these CVs show the polarization between the oxidation and reduction peaks to be very small for each compound, even for CV at 2 mV s^−1^. This result suggests that the intercalation process is not limited severely by low ionic diffusion, a consideration which will be discussed later.

CVs at different scan rates were performed to assess the capacity retention as a function of increasing scan rate. Scan rates from 0.1 mV s^−1^ to 50 mV s^−1^ were chosen, corresponding to 5.5h and 40s of charge, respectively. Figures 4C,D shows the specific capacity and relative capacity of every material at the mentioned scan rates. Ag_0.40_La_0.20_□_0.40_NbO_3_, the material with the highest number of vacancies, provides the highest capacity at a low sweep rate (Figure 4C). However, when the scan rate is increased, a rapid decrease in capacity is observed starting at 0.2 mV s^−1^. As shown in Figure 4D, at 50 mV s^−1^, Ag_0.40_La_0.20_□_0.40_NbO_3_ retains only 34% of the initial capacity measured at 0.1 mV s^−1^. Similarly, Ag_0.55_La_0.15_□_0.30_NbO_3_ demonstrates a relatively high capacity at low scan rates but a rapid drop at higher rates. In contrast, while less substituted materials such as Ag_0.85_La_0.05_□_0.10_NbO_3_ and Ag_0.70_La_0.10_□_0.20_NbO_3_ exhibit lower capacities at 0.1 mV s^−1^, they demonstrate better capacity retention. At 50 mV s^−1^, Ag_0.85_La_0.05_□_0.10_NbO_3_, and Ag_0.70_La_0.10_□_0.20_NbO_3_ exhibit capacity retentions of 53 and 64%, respectively, for a 500 times faster rate of charge/discharge compared to their capacities measured at 0.1 mV s^−1^. All the specific capacities are summarized in Supplementary Table S4.

**FIGURE 5:**
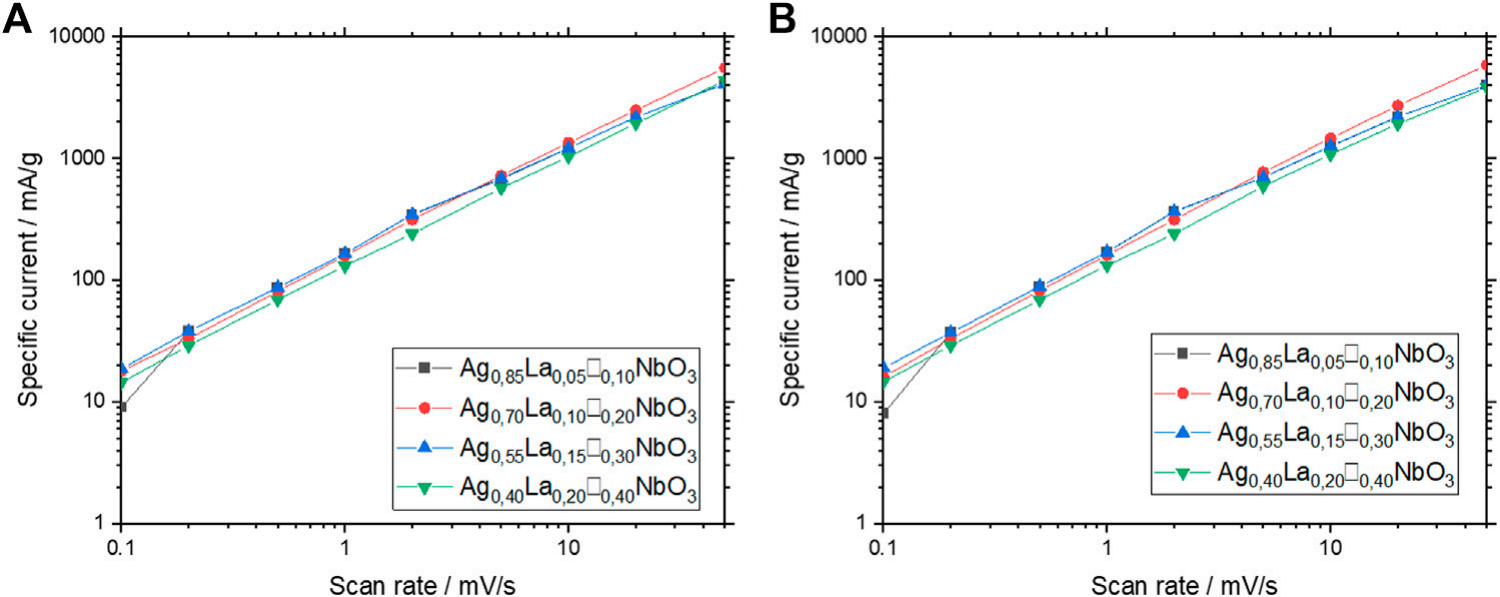
**(A)** Linear regression from power law in the reduction peaks of Ag_1-3x_La_x_□_2x_NbO_3_ for different value of x. **(B)** Linear regression from power law in the oxidation peaks of Ag_1-3x_La_x_□_2x_NbO_3_ for different value of x.

In order to get a better understanding of the charge storage mechanism, *b*-values from the power law relationship in Eq. 3 have been calculated,

$$\mathrm{i=}a.v^{b}$$ (3)

where i is the intensity of reduction/oxidation peak, v the corresponding scan rate, *a* and *b* are constant values. If *b* is close to 0.5, charge storage can be described as limited by ionic diffusion in the host material, as described in the Randles-Ševčík equation (Ševčík 1948; Randles 1948). When the value of *b* is equal or close to 1, charge storage occurs in agreement with non-diffusion limited mechanism, as in the case of pseudocapacitive materials (Choi et al., 2020). Regarding Ag_1-3x_La_x_□_2x_NbO_3_ compounds, as shown in Figure 5A,B, a linear relationship between specific current and scan rate (0.1 mV s^−1^ and 50 mV s^−1^) is observed, the *b*-values of which are listed in Table 1. The values of *b* vary between 0.87 and 0.95, which seems to indicate weak diffusion limitation during charge/discharge processes. Additionally, the smaller *b* values calculated for Ag_0.55_La_0.15_□_0.30_NbO_3_ and Ag_0.40_La_0.20_□_0.40_NbO_3_ support the difference in capacity retention observed previously, as compared to Ag_0.85_La_0.05_□_0.10_NbO_3_, and Ag_0.70_La_0.10_□_0.20_NbO_3_ *.*

**TABLE 1:** *b* value of Ag_1-3x_La_x_□_2x_NbO_3_ for different value of x for reduction and oxidation peaks.

	Reduction	Oxydation
Ag_0.85_La_0.05_□_0.10_NbO_3_	0.94	0.95
Ag_0.70_La_0.10_□_0.20_NbO_3_	0.92	0.95
Ag_0.55_La_0.15_□_0.30_NbO_3_	0.87	0.87
Ag_0.40_La_0.20_□_0.40_NbO_3_	0.9	0.91

**FIGURE 6:**
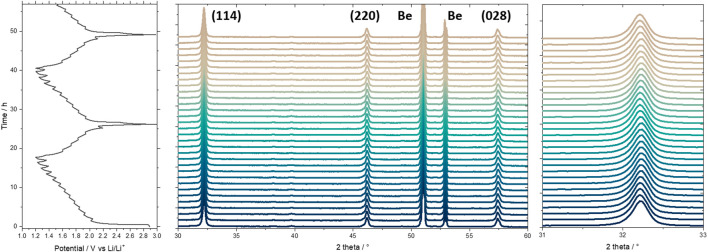
*In situ* XRD patterns of the Ag_0.70_La_0.10_□_0.20_NbO_3_ at a current of 0.02 A g^−1^ with corresponding voltage-time curve.

As displayed in Figure 6, the behavior of the different oxides during lithium insertion was investigated by *in situ* XRD, to better understand the filling of the vacant A-sites of the Ag_1-3x_La_x_□_2x_NbO_3_ perovskite. Ag_0.70_La_0.10_□_0.20_NbO_3_ was used because no structural defects were observed in this phase compared to Ag_0.55_La_0.15_□_0.30_NbO_3_ and Ag_0.40_La_0.20_□_0.40_NbO_3_. First, no new peak appears (and/or disappears) on the XRD patterns during charge and discharge. This behavior indicates that a solid solution type mechanism predominates upon charge storage, as previously reported for intercalation materials dedicated to power application with a *b* value close to 1 (Come et al., 2014). Moreover, it is observed that the peaks initially present do not move during lithiation. The three diffraction peaks observed in the studied 2θ range present the three crystallographic directions, implying that the perovskite structure does not modify its lattice parameters during lithium cation insertion in the vacant A sites. Thus, this material can be considered as a zero strain oxide during lithium insertion, similar to the well-known Li_4_Ti_5_O_12_ (Thackeray and Amine 2021).

**FIGURE 7:**
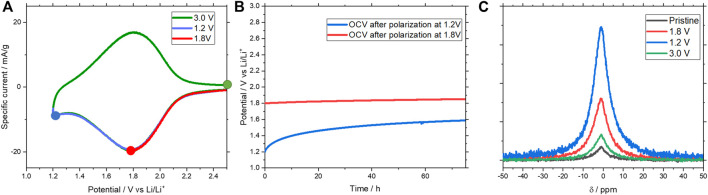
**(A)** CV, **(B)** Self-discharge curves and **(C)** *ex situ* Li^7^ MAS NMR normalized spectrum of the Ag_0.70_La_0.10_□_0.20_NbO_3_ at different state of charge/discharge with associated CVs.

In another series of measurements, self-discharge and NMR experiments carried out on cycled Ag_0.70_La_0.10_□_0.20_NbO_3_ electrodes provide further evidence of Li^+^ insertion in the material. For these experiments, electrodes were cycled to 1.8 and 1.2 V vs. *Li* ^*+*^ */Li* at 0.1 mV s^−1^ and subsequently polarized for 10 h to reach a thermodynamic equilibrium. As shown in Figure 7A,B, both electrodes (cycled at 1.2 and 1.8 V vs. *Li* ^*+*^ */Li*) present relatively low self-discharge. The electrode polarized at 1.8V shows a potential of 1.82 V vs. *Li* ^*+*^ */Li* after almost 80 h of OCV. The electrode polarized at 1.2 V shows a potential of 1.59 V *vs. Li* ^*+*^ */Li* in the same condition. These low self-discharge levels at 1.2 and 1.8 V are a strong indication that lithium cations are inserted in a stable way into the vacancies of the material. *Ex situ* ^7^Li MAS NMR measurements have been performed after these self-discharge experiments. ^7^Li MAS NMR spectra were also recorded for the pristine and the fully discharged electrode (3.0V) in addition to the charged samples at 1.2 V vs. *Li* ^*+*^ */Li*, at a similar scan rate of 0.1 mV s^−1^. As expected, the pristine electrode has a negligible quantity of Li detected. For the partially and fully charged samples, a peak is observed at around −1 ppm. The integrated intensity of the NMR peak, which is intrinsically proportional to the detected amount of lithium in the materials, is also found to be proportional to the specific capacity of the materials at 1.8 and 1.2 V (Figure 7B,C). The spectrum of the fully discharged electrode at 3.0V displays a small amount of lithium assigned to the partial irreversibility of the reaction during the first cycle and is also found to be in agreement with the first cycle of the CV. Due to the amount of lithium inserted in the materials, the signal to noise ratio remains relatively low even for a large number of scans. For this reason, spectra with a narrower linewidth and thus better resolution, were not possible. Thus, it is difficult to conclude as to the number of lithium sites, even at the fully charged state, as only one apparent resonance is observed in ^7^Li MAS NMR. Taken together, the combination of *ex situ* NMR and self-discharge provide convincing evidence that lithium ions are inserted in this perovskite material, most probably in the vacant A site.

**FIGURE 8:**
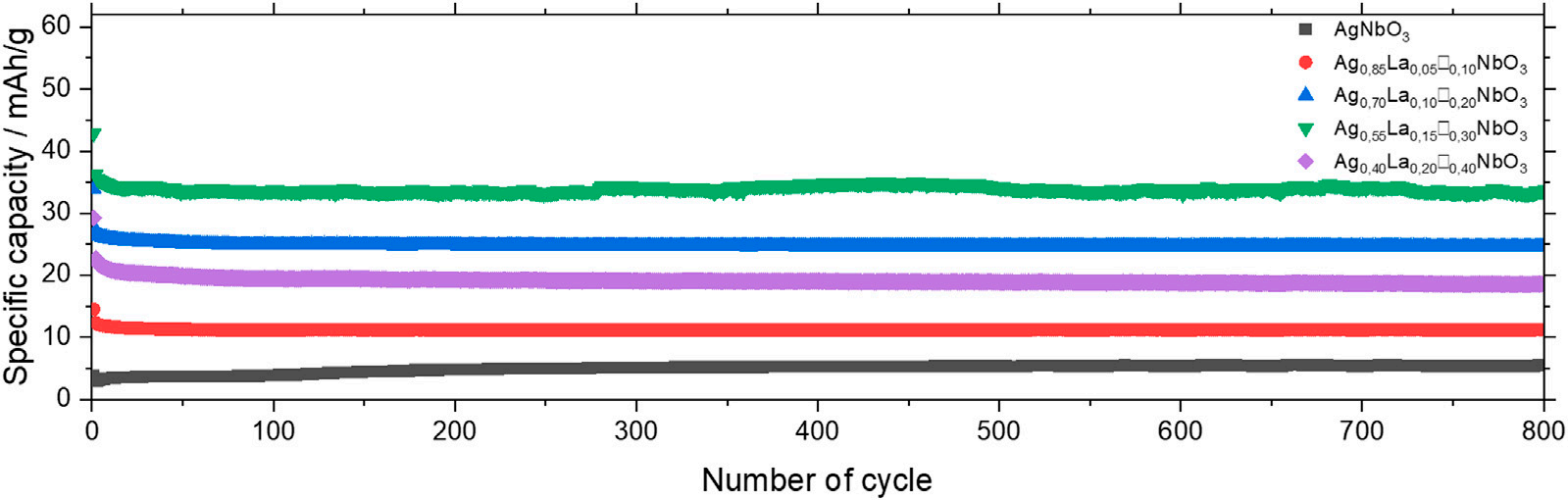
Cycling stability of Ag_1-3x_La_x_□_2x_NbO_3_ at 2 mV s^−1^

The cycling characteristics for Ag_1-3x_La_x_□_2x_NbO_3_ were determined as cycle life is a key property for the development of batteries based on high rate electrode materials. As observed in Figure 8, all the materials exhibit exceptional stability upon cycling. After 800 cycles at 2 mV s^−1^, Ag_0.85_La_0.05_□_0.10_NbO_3_, Ag_0.70_La_0.10_□_0.20_NbO_3_, Ag_0.55_La_0.15_□_0.30_NbO_3_ and Ag_0.40_La_0.20_□_0.40_NbO_3_ show a capacity retention of 95, 95, 97 and 88%, respectively. The *in situ* XRD experiments and *b*-value calculation, which show zero-strain materials and non-diffusion limited charge storage mechanisms, respectively, are consistent with the outstanding cyclability observed for these materials.

In contrast to the classical design of insertion materials, our approach was to synthesize an oxide with vacancies in order to create potential sites for cation insertion in a perovskite. As proven by *ex situ* MAS NMR, lithium ions can be reversibly stored in the A site of Ag_0.70_La_0.10_□_0.20_NbO_3_ and by extension to every Ag_1-3x_La_x_□_2x_NbO_3_ composition. To further demonstrate this point, Supplementary Figure S3A–D presents the difference between lithium and sodium insertion by cyclic voltammetry at different scan rates. While Li^+^ insertion leads to characteristic redox peaks, in the case of Na^+^, no redox reaction can be observed. It is likely that sodium ions cannot be inserted into the structure of Ag_0.70_La_0.10_□_0.20_NbO_3_ and therefore cannot generate redox reactions due to the larger ionic radius This is also the case for the pseudocapacitive T-Nb_2_O_5,_ which shows highly reversible Li-ion intercalation but negligible Na-ion insertion (Kim et al., 2012). Moreover, kinetic analysis provides compelling evidence that a not diffusion-limited insertion occurs during the charge and discharge processes.

## Conclusion

This work presents the partial substitution of AgNbO_3_ perovskite by lanthanum in the A-site, resulting in the creation of cationic vacancies. We report on a gradual increase of the capacity at low scan rates as a function of the increase of the substitution. In addition, the different oxides present excellent behavior at high scan rates. At 50 mV.s^−1^Ag_0.70_La_0.10_□_0.20_NbO_3_ retains 64% of its capacity measured at 0.1 mV s^−1^. Moreover, the combination of *ex situ* MAS NMR and *in situ* XRD experiments confirm that the insertion of lithium ions in the A site of the perovskite is responsible for the observed capacity and does not cause any structural change of the material. Together, these observations help to explain the good cycling behavior observed. In addition to structural insight, kinetic analysis indicates a non-diffusion limited ion insertion process for these oxides.

Nevertheless, some questions still remain. A better understanding of the solvation/desolvation occurring at the surface of particles during alkali ion insertion is critical towards understanding the small limitation of diffusion in charge storage process (Wang et al., 2019). Further experiments such as EQCM or *operando* atomic force microscopy can provide more insight in the charge storage mechanism. From another point of view, the promising observations reported here should be explored with other vacancy-designed materials and with different alkali cations. Moreover, these oxides present a charge storage mechanism which are between double layer capacitor and battery type electrodes (as observed for MnO_2_ (Toupin et al., 2004; Brousse et al., 2006), T-Nb_2_O_5_ (Augustyn et al., 2013; Griffith et al., 2016) or MXenes (Naguib et al., 2012; Anasori et al., 2017) and represent an interesting model material for fundamental understanding in general.

In conclusion, the new approach reported in this work presents a different type of material design which widens the opportunities for creating the next generation of negative electrodes for high power Li- ion batteries. Although the oxide presented here contains silver, we believe this study can open the way toward the search of more practical materials for high-power battery electrodes.

## Data Availability

The raw data supporting the conclusion of this article will be made available by the authors, without undue reservation.
