# Supplementary material for: Investigating the Perovskite Ag1-3xLaxNbO3 as a High-Rate Negative Electrode for Li-Ion Batteries
Source: Front Chem. 2022 Apr 13;10:873783. doi: 10.3389/fchem.2022.873783 (PMC9043289; doi:10.3389/fchem.2022.873783)
Supplement: Supplementary file 1 [file DataSheet1.PDF]

Supplementary Material

Supplementary Table 1. Specific surface area of $Ag_{1-3x}La_{x}\square_{2x}NbO_3$ for different values of x.

		Specific surface area / $m^2.g^{-1}$
x= 0	$AgNbO_3$	3 $m^2.g^{-1}$
x= 0.05	$Ag_{0.85}La_{0.05}\square_{0.10}NbO_3$	6 $m^2.g^{-1}$
x= 0.10	$Ag_{0.70}La_{0.10}\square_{0.20}NbO_3$	13 $m^2.g^{-1}$
x= 0.15	$Ag_{0.55}La_{0.15}\square_{0.30}NbO_3$	15 $m^2.g^{-1}$
x= 0.20	$Ag_{0.40}La_{0.20}\square_{0.40}NbO_3$	16 $m^2.g^{-1}$

Supplementary Table 2. Experimental obtain by EDX (in bold) and theoretical percentage of Ag, La and Nb of $Ag_{1-3x}La_{x}\square_{2x}NbO_3$ for different values of x.

	%Ag	%La	%Nb
$AgNbO_3$	**49**/50	**0**/0	**51**/50
$Ag_{0.85}La_{0.05}\square_{0.10}NbO_3$	**40**/44	**5**/3	**55**/33
$Ag_{0.70}La_{0.10}\square_{0.20}NbO_3$	**33**/39	**8**/6	**59**/55
$Ag_{0.55}La_{0.15}\square_{0.30}NbO_3$	**30**/32	**11**/9	**59**/59
$Ag_{0.40}La_{0.20}\square_{0.40}NbO_3$	**26**/25	**13**/12.5	**61**/62.5

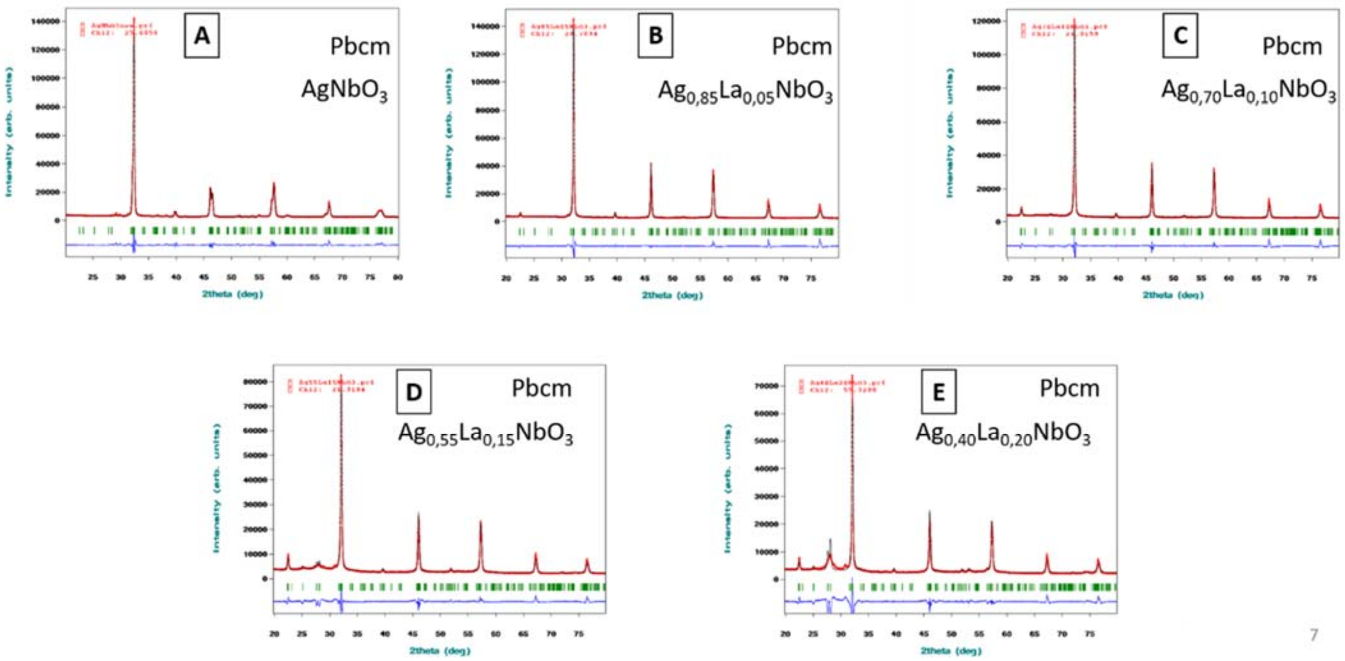

Supplementary Figure 2. Le Bail refinement using the space group Pbcm of (a) $AgNbO_3$ (b) $Ag_{0.85}La_{0.05}\square_{0.10}NbO_3$ (c) $Ag_{0.70}La_{0.10}\square_{0.20}NbO_3$ (d) $Ag_{0.55}La_{0.15}\square_{0.30}NbO_3$ (e) $Ag_{0.40}La_{0.20}\square_{0.40}NbO_3$

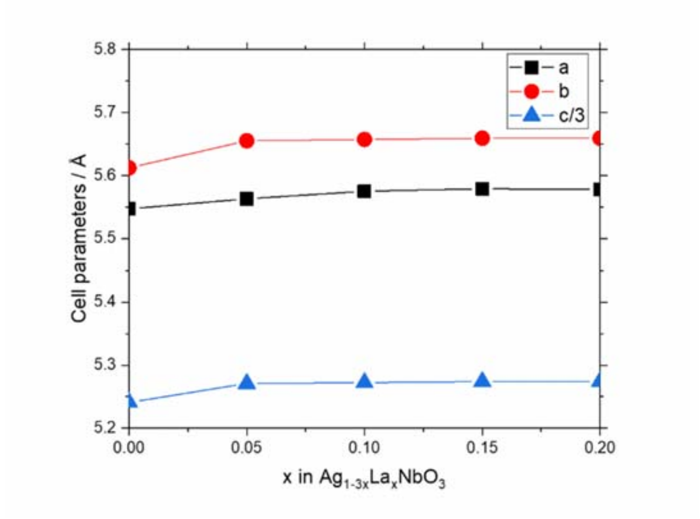

Supplementary Figure 2. (a) Cell parameters of $Ag_{1-3x}La_x\square_{2x}NbO_3$ for different value of x.

Supplementary Table 3. Cell parameters of $Ag_{1-3x}La_x\square_{2x}NbO_3$ for different values of x.

	a / A°	b / A°	c / A°	Volume / A°^{3}
$AgNbO_3$	5.547	5.612	15.723	489.453
$Ag_{0.85}La_{0.05}\square_{0.10}NbO_3$	5.563	5.655	15.813	497.4557
$Ag_{0.70}La_{0.10}\square_{0.20}NbO_3$	5.575	5.657	15.817	498.833
$Ag_{0.55}La_{0.15}\square_{0.30}NbO_3$	5.579	5.659	15.822	499.525
$Ag_{0.40}La_{0.20}\square_{0.40}NbO_3$	5.578	5.659	15.822	499.436

Supplementary Table 4. Specific capacity for every material at different scan rate during CV experiments.

	$AgNbO_3$	$Ag_{0.85}La_{0.05}\square_{0.10}NbO_3$	$Ag_{0.70}La_{0.10}\square_{0.20}NbO_3$	$Ag_{0.55}La_{0.15}\square_{0.30}NbO_3$	$Ag_{0.40}La_{0.20}\square_{0.40}NbO_3$
**0.1 mV.s^{-1}**	**6.0**	**17.0**	**32.2**	**34.2**	**41.1**
**0.2 mV.s^{-1}**	**5.9**	**12.1**	**26.6**	**34.0**	**27.7**
**0.5 mV.s^{-1}**	**5.9**	**11.6**	**25.8**	**32.3**	**25.3**
**1.0 mV.s^{-1}**	**5.7**	**11.5**	**25.6**	**31.0**	**24.1**
**2.0 mV.s^{-1}**	**4.8**	**11.0**	**25.1**	**31.0**	**21.9**
**5.0 mV.s^{-1}**	**4.6**	**10.9**	**24.6**	**25.9**	**19.7**
**10.0 mV.s^{-1}**	**3.8**	**10.6**	**24.0**	**23.3**	**19.7**
**20.0 mV.s^{-1}**	**3.1**	**10.1**	**23.2**	**20.2**	**17.5**
**50.0 mV.s^{-1}**	**2.3**	**9.1**	**20.7**	**15.2**	**13.9**

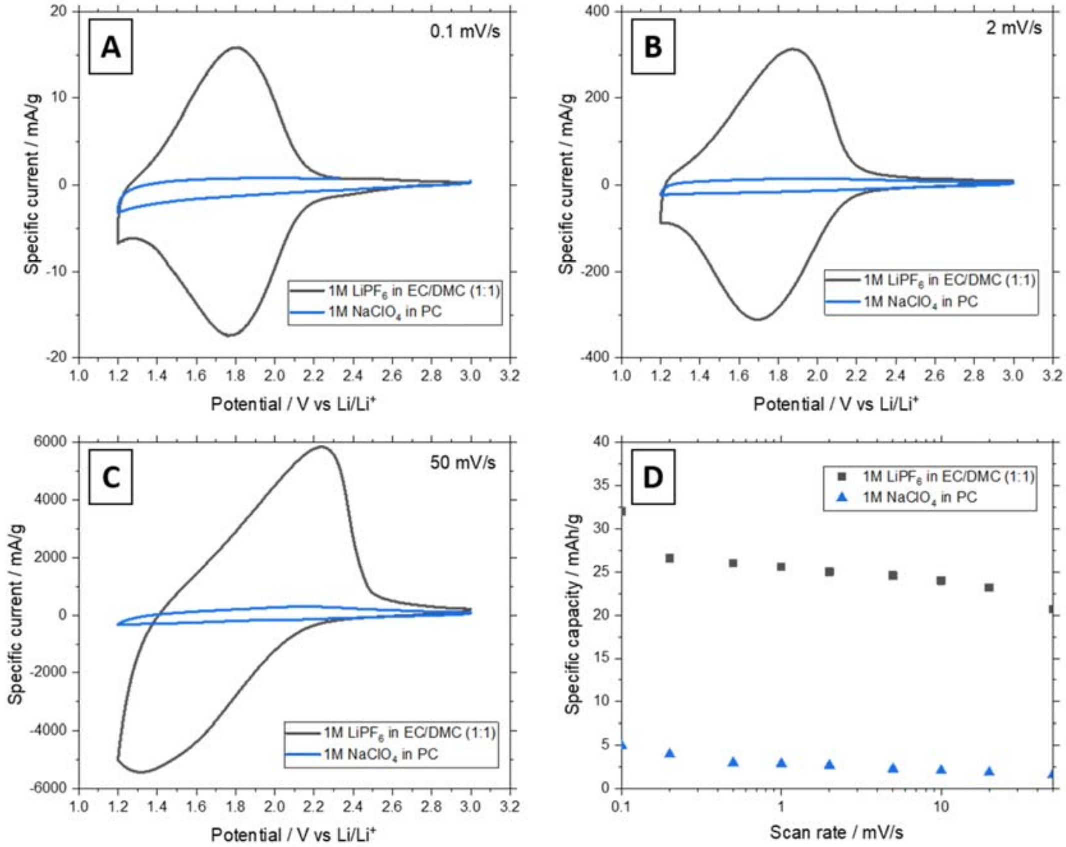

Supplementary Figure 3. (a-b-c) CV at 0.1 mV.s^{-1}, 2 mV.s^{-1}, 50 mV.s^{-1} respectively between 1.2V and 3V of $Ag_{0.70}La_{0.10}\square_{0.20}NbO_3$. . (d) Specific capacity of $Ag_{0.70}La_{0.10}\square_{0.10}NbO_3$ for different type of electrolytes. (1M $LiPF_6$ in EC/DMC (1:1) and 1M $NaClO_4$ in PC (1:1).
